# Evaluating the Therapeutic Mechanisms of Selected Active Compounds in Cornus Officinalis and Paeonia Lactiflora in Rheumatoid Arthritis via Network Pharmacology Analysis

**DOI:** 10.3389/fphar.2021.648037

**Published:** 2021-04-21

**Authors:** Qinglin Li, Shaoqi Hu, Lichuang Huang, Jida Zhang, Gang Cao

**Affiliations:** ^1^ Scientific Research Department, The Cancer Hospital of the University of Chinese Academy of Sciences (Zhejiang Cancer Hospital), Hangzhou, China; ^2^ College of Pharmaceutical Sciences, Zhejiang Chinese Medical University, Hangzhou, China; ^3^ Institute of Basic Research in Clinical Medicine, College of Basic Medical Science, Zhejiang Chinese Medical University, Hangzhou, China

**Keywords:** traditional chinese medicine, collagen-induced arthritis, compound-target relationship, gene ontology, KEGG, molecular docking

## Abstract

Cornus officinalis Sieb et. Zucc and Paeonia lactiflora Pall. have exhibited favorable therapeutic effects against rheumatoid arthritis (RA), but the specific mechanisms of their active compounds remain unclear. The aim of this study was to comprehensively analyze the therapeutic mechanisms of selected active compounds in Cornus officinalis (loganin, ursolic acid, and morroniside) and Paeonia lactiflora (paeoniflorin and albiflorin) via network pharmacology. The pharmacological properties of the five active compounds were evaluated and their potential target genes were identified by database screening. Gene Ontology and Kyoto Encyclopedia of Genes and Genomes functional analysis were performed to determine the enriched molecular pathways associated with the active compounds. Using network pharmacology tools, eight genes (IL1β, VEGFA, STAT3, TP53, IL6, TNF, FOS, and LGALS3) were identified as common targets between RA and the five active compounds. Molecular docking simulation revealed the compound-target relationship between the five active compounds and three selected targets from the eight common ones (LGALS3, STAT3, and VEGFA). The compound-target relationships were subsequently validated via preliminary *in vivo* experiments in a rat model of collagen-induced arthritis. Rats subjected to collagen-induced arthritis showed increased protein expression of LGALS3, STAT3, and VEGFA in synovial tissues. However, treatment using Cornus officinalis or/and Paeonia lactiflora, as well as their most drug-like active compounds (ursolic acid or/and paeoniflorin, respectively, identified based on pharmacological properties), attenuated the expression of these three targets, as previously predicted. Collectively, network pharmacology allowed the pharmacological and molecular roles of Cornus officinalis and Paeonia lactiflora to be systematically revealed, further establishing them as important candidate drugs in the treatment and management of RA.

## Introduction

Rheumatoid arthritis (RA) is a chronic autoimmune disease that mainly affects the joints and causes pain and stiffness. This occurs via a self-attack mechanism within the body’s immune system that targets the joints (Gierut et al., 2010; Bolon, 2012), leading to synovial inflammation, thickening of the joint capsule, and damage to surrounding bone and cartilage (Sweeney and Firestein, 2004; Sudol-Szopinska et al., 2017; Ostrowska et al., 2018). The etiology of RA is unclear, but it is believed that both genetic and environmental factors contribute to its initiation and development (Deane et al., 2017). RA treatment and management mainly aim to alleviate physical pain and inflammation via therapeutic exercise and the use of assistive devices (Kavuncu and Evcik, 2004). In some cases, disease-modifying anti-rheumatic drugs such as hydroxychloroquine and leflunomide may be applied to slow the progression of RA, but the use of these drugs may result in a variety of adverse effects (Deng et al., 2020). Surgery is another method of managing RA that aims to repair or fuse joints and may help in certain situations. The popularity of alternative and complementary medicine has grown substantially in recent years, but its effectiveness remains to be validated.

Traditional Chinese medicine (TCM) is a form of alternative and complementary medical practice that has attracted interest from medical researchers in recent decades. The most common TCM drugs and compounds are natural or herbal substances that have the advantage of lowered side effects. The main feature of TCM is its multi-target characteristic, meaning that medical compounds are composed of a variety of biologically active components that each may target different symptoms. This unique characteristic has accentuated the potential of TCM in the treatment of complex diseases such as RA, which is associated with both inflammatory and immune anomalies. Among herbal substances used in TCM, Cornus officinalis Sieb. et Zucc (or Corni Fructus) and Paeonia lactiflora Pall. are known to exhibit anti-inflammatory and immunomodulatory effects. Cornus officinalis is a dogwood species native to East Asian countries such as China, Korea, and Japan. It contains approximately 90 identified compounds including iridoids (loganin, morronisides, cornusfurosides, etc.), secoiridoids (linalool, secoxyloganin, etc.), triterpenoids (ursolic acid, oleanolic acid, and arjunglucoside II), and flavonoids (kaempferol, kaempferide, quercetin, etc.) (Dong et al., 2018). Studies have revealed that Cornus officinalis exhibited anti-inflammatory, anti-allergic, and anti-oxidant properties in the treatment of atopic dermatitis (Quah et al., 2020) and anti-neoplastic effects against hepatocellular carcinoma (Chang et al., 2004). Paeonia lactiflora is an herbaceous perennial flowering plant in the Paeoniaceae family composed of compounds including paeoniflorin, albiflorin, and oxypaeoniflorin. The isomers paeoniflorin and albiflorin have shown, in addition to their anti-inflammatory activities (Wang et al., 2014), therapeutic effectiveness against neuropathic pain (Zhou et al., 2016) and bone marrow suppression (Zhu et al., 2016). Given this, the effect of Cornus officinalis and Paeonia lactiflora on RA and the molecular mechanisms underlying the action of their constituents remain to be investigated.

Network pharmacology combines systems biology with biological network construction and analysis to assess the effectiveness and metabolic characteristics of drugs (Huang et al., 2014). It takes advantage of information networks based on high-throughput omics data analysis, virtual computing, and database retrieval (Wang et al., 2020). In particular, it has been applied in elucidating the synergistic multi-component, multi-target, and multi-pathway effect of TCM prescriptions and in clarifying drug action mechanism. Molecular docking is a technical means of discovering new drugs that applies computer-aided design to simulate the force and structure of molecules through chemometrics. It is performed by searching for low-energy binding modes between small molecules (ligands) and macromolecules with known structures (receptors) at the active site (Azam and Abbasi, 2013). The use of network pharmacology to build multi-level network models has become a strategy to scientifically evaluate the effectiveness of TCM.

For this study, a comprehensive literature survey was performed to compare the active compounds of Cornus officinalis and Paeonia lactiflora. Based on criteria such as relative content, oral bioavailability, and solubility, three active compounds of Cornus officinalis (loganin, ursolic, and morroniside) were selected for investigation in this study. Furthermore, the isomers paeoniflorin and albiflorin were included as the active compounds of Paeonia lactiflora. The targets and mechanisms of these five compounds were explored via network pharmacology and molecular docking and preliminarily verified in a rat model of collagen-induced arthritis (CIA).

## Materials and Methods

### Predictive Screening of the Target Genes of Selected Compounds

The target proteins of loganin, ursolic acid, morroniside, paeoniflorin, and albiflorin were screened using three databases. First, targets were screened using the Traditional Chinese Medicine System Pharmacology (TCMSP) database and Analysis Platform (https://www.tcmspw.com/tcmsp.php). Based on the predicted target proteins corresponding to the chemical small molecules identified by TCMSP, the names of the target proteins were matched with the abbreviated gene symbols corresponding to the uniprotID in the uniport database (https://www.uniprot.org). Next, targets were screened using the PharmMapper database (http://www.lilab-ecust.cn/pharmmapper) based on the chemical small molecules identified in the database. Finally, targets were screened using the Swiss database (http://www.swisstargetprediction.ch) based on the chemical small molecules identified in the database. All predicted targets from TCMSP and the top ten targets from PharmMapper and Swiss were selected, and a Venn diagram was generated to identify overlapping targets between databases. In total, 132 unique gene targets of the five compounds were identified between the three databses.

### Gene Ontology and Kyoto Encyclopedia of Genes and Genomes Functional Analysis

GO and KEGG functional analysis of the previously screened targets was performed using the database for Annotation, Visualization, and Integrated Discovery website (https://david.ncifcrf.gov). *p* ≤ 0.05 was defined as the critical value of significant enrichment, and results were mapped using R software as bar plots and bubble plots for both GO and KEGG analysis. The “Pathview” package in R software was used to generate a diagram of signaling pathways associated with RA based on the results of KEGG analysis.

### Protein-Protein Interaction Analysis of Target Interactions

The interactions among the target proteins of the selected compounds was mapped by constructing a PPI network to present the direct and indirect regulatory relationship between the targets, using the STRING web site (https://string-db.org). For visual analysis of the target proteins, a visual PPI network diagram was generated using Cytoscape 3.6.1, and the Network Analyzer function in Cytoscape was utilized to evaluate the topology parameters of the network nodes. In addition, compound-target relationships were visualized by constructing a pharmacological network map using Cytoscape.

### Identification of Common Targets of Rheumatoid Arthritis and Active Compounds

The potential targets of RA were screened in the following databases: OMIM (https://www.omim.org), GenCLiP3 (http://ci.smu.edu.cn/genclip3/analysis.php), CTD (http://ctdbase.org), and GeneCards (https://www.genecards.org). The targets of RA identified here were illustrated using a Venn diagram, revealing 71 common targets between the four databases (Supplementary Table S1). Then, the 71 targets of RA were compared with those of the five selected active compounds to reveal the common targets.

### Molecular Docking Analysis

Based on the active compounds and core targets selected above, the protein structure of the corresponding target was obtained from the PDB database (http://www.rcsb.org/). Molecular docking analysis was performed using the AutoDock Vina software (http://vina.scripps.edu/) (Trott and Olson, 2010).

### Collagen-Induced Arthritis Induction and Drug Treatment

Commercially available Cornus officinalis (COR) and Paeonia lactiflora (PAE) were purchased from Zhejiang Chinese Medical University Medical Pieces Ltd. (Hangzhou, China), ursolic acid (UA, DX0019) and paeoniflorin (PF, DS0070) were purchased from Desite (Chengdu, China), and dexamethasone (DEX, D1756) was purchased from Sigma-Aldrich (St. Louis, MO). Each compound was dissolved in sterilized water, given that drug administration is performed at 10 ml per kg of animal body weight. All animal experiments were performed at Wuhan Myhalic Biotechnology Co., Ltd. (Wuhan, China). The experimental protocol was approved by the institutional review board of the Model Animal Research Institute at Wuhan Myhalic Biotechnology Co., Ltd. and adhered to the guidelines for animal care and use (approval number: HLK-20190418–01). Male specific-pathogen-free Sprague-Dawley rats weighing 200 ± 20 g, acquired from China Three Gorges University, were housed in a facility with 50–60% relative humidity at 25°C. Before the experiment, the rats adaptively fed for 7 days, where they were allowed free access to food and water. The CIA model was established following previously reported methods (Jia et al., 2014), with three rats in each group. Control rats were not treated in any way. To induce CIA, 10 mg of type II collagen (PAB43878, Bioswamp) was mixed with 0.01 M acetic acid and emulsified with an equal volume of Freund’s complete adjuvant to a final collagen concentration of 2 mg/ml. On day 0, experimental rats were intradermally injected with 0.1 ml of emulsified collagen II in the right hind toe. Boost immunization was induced after 7 days with an additional intradermal injection of 0.1 ml of emulsified collagen II in the right hind toe. Drug treatment began after another 7 days (14 days of CIA induction) by gavaging the rats daily with the following drug doses: COR: 3.36 g of drug per kg of body weight per day (g/kg/d); PAE: 6.27 g/kg/d; UA: 25 mg/kg/d; PF: 7.5 mg/kg/d; DEX (positive control): 0.5 mg/kg/d.

### Sample Preparation

After 20 days of drug administration (34 days after initial CIA induction), the rats were sacrificed via an overdose of sodium pentobarbital. Synovial tissues were isolated by cutting open the knee joint to expose the kneecap and separating the muscles. The synovial and fibrous layers of the joint capsule were separated using surgical scalpels, and synovial tissues were extracted. The right ankle joint was fixed in 4% paraformaldehyde, decalcified, and paraffin-embedded for histological examination.

### Immunohistochemistry

Tissue sections were heated at 65°C for 1 h and immersed in xylene for 30 min. After deparaffinization, the sections were washed in tap water for 10 min. Antigen retrieval was performed by immersing the sections in 0.01 M sodium citrate buffer at 125°C and 103 kPa for 20 min, and the sections were then washed three times with phosphate-buffered saline (PBS) after cooling. Endogenous peroxidase was performed in 3% H_2_O_2_ at 37°C for 10 min and the sections were thereafter washed three times with PBS. The sections were then blocked in 10% goat serum (SL038, Solarbio) at 37°C for 20 min in a humidified atmosphere and washed three times with PBS. For staining, the sections were incubated overnight at 4°C with primary antibodies against galectin-3 (LGALS3, PAB32080, Bioswamp, Wuhan, China), signal transducer and activator of transcription 3 (STAT3, PAB30641, Bioswamp), and vascular endothelial growth factor-A (VEGFA, PAB45842, Bioswamp) (all diluted at 1:50 in PBS). Thereafter, the sections were washed three times with PBS and incubated for 1 h at 37°C with secondary antibodies in the MaxVision™ HRP-Polymer anti-Mouse/Rabbit IHC Kit (KIT-5020, Maxim, Fuzhou, China). After three washes with PBS, the sections were stained with diaminobenzidine, washed with tap water, and counterstained with hematoxylin (G1140, Solarbio) for 3 min. After ethanol dehydration and xylene washes, the sections were sealed and observed under a microscope. Brown areas represent positive staining.

### Data Analysis

The intensity of positive immunohistochemical staining was quantified by ImagePro Plus using three sections from each treatment group (*n* = 3). The data are represented as the average integrated optical density ± standard deviation. One-way analysis of variance with Tukey’s post-hoc analysis was performed to evaluate the differences among groups. *p* < 0.05 was considered as statistically significant.

## Results

### Molecular and Drug-Like Properties of Active Compounds

**FIGURE 1:**
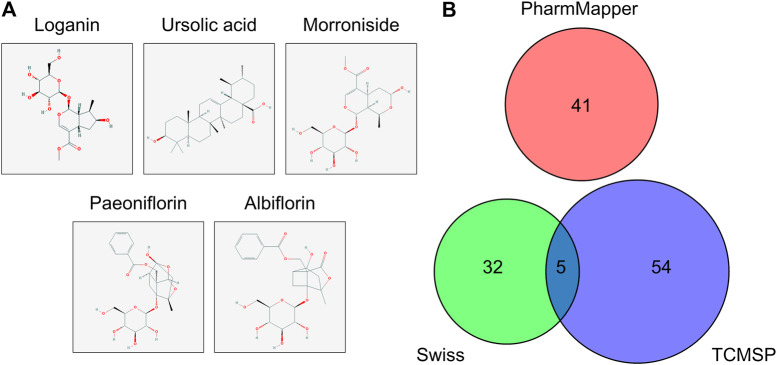
Molecular structure and potential targets of the active compounds of Cornus officinalis and Paeonia lactiflora. **(A)** Three active compounds of Cornus officinalis (loganin, ursolic acid, and morroniside) and two of Paeonia lactiflora (paeoniflorin and albiflorin) were examined in this study. **(B)** In total, 132 target genes among the five active compounds were identified by three databases (Swiss, TCMSP, and PharmMapper), with five targets overlapping between Swiss and PharmMapper. However, no target gene was commonly identified by all three databases.

The molecular structures of the selected active compounds of Cornus officinalis (loganin, ursolic acid, and morroniside) and Paeonia lactiflora (paeoniflorin and albiflorin) investigated in this study are shown in Figure 1A. The “absorption, distribution, metabolism, and excretion” (ADME)-related pharmacological parameters of these compounds are listed in Table 1, and the detailed descriptions of these parameters can be found on the TCMSP web site (https://tcmspw.com/load_intro.php?id=29). The “drug-likeness” of a compound can be evaluated from these parameters by referring to Lipinski’s “Rule of 5”, which is a guide to determining whether a compound’s pharmacological activity is likely to render it orally active in humans (Lipinski et al., 2001). Among the five compounds, ursolic acid is the only one that satisfies three out of the four criteria in the Rule of 5: Hdon < 5, Hacc < 10, and MW < 500. In addition, paeoniflorin exhibits the highest OB (oral bioavailability) score of 53.87% and the highest DL (drug-likeness) score of 0.79. These results signify that among the five active compounds, ursolic acid and paeoniflorin (active compounds in Cornus officinalis and Paeonia lactiflora, respectively) exhibited the most “drug-like” pharmacological properties.

**TABLE 1:** Pharmacological and molecular properties of active compounds investigated in this study.

Compound	Loganin	Ursolic acid	Morroniside	Paeoniflorin	Albiflorin
**Molecule ID**	MOL007004	MOL000511	MOL001683	MOL001924	MOL007004
**MW (g/mol)**	390.43	456.78	406.43	480.51	480.51
**AlogP**	−2.08	6.47	−2.47	−1.28	−1.33
**Hdon**	5	2	5	5	5
**Hacc**	10	3	11	11	11
**OB (%)**	5.9	16.77	13.86	53.87	30.25
**Caco-2**	−1.48	0.67	−2.01	−1.47	−1.52
**BBB**	−2.26	0.07	−5.11	−1.86	−2.33
**DL**	0.44	0.75	0.5	0.79	0.77
**FASA-**	0.24	0.26	0.25	0.34	0.35
**TPSA**	155.14	57.53	164.37	164.37	172.21
**RBN**	5	1	5	7	7

### Target Prediction of Active Compounds and Gene Ontology Functional and Kyoto Encyclopedia of Genes and Genomes Pathway Enrichment Analysis

**FIGURE 2:**
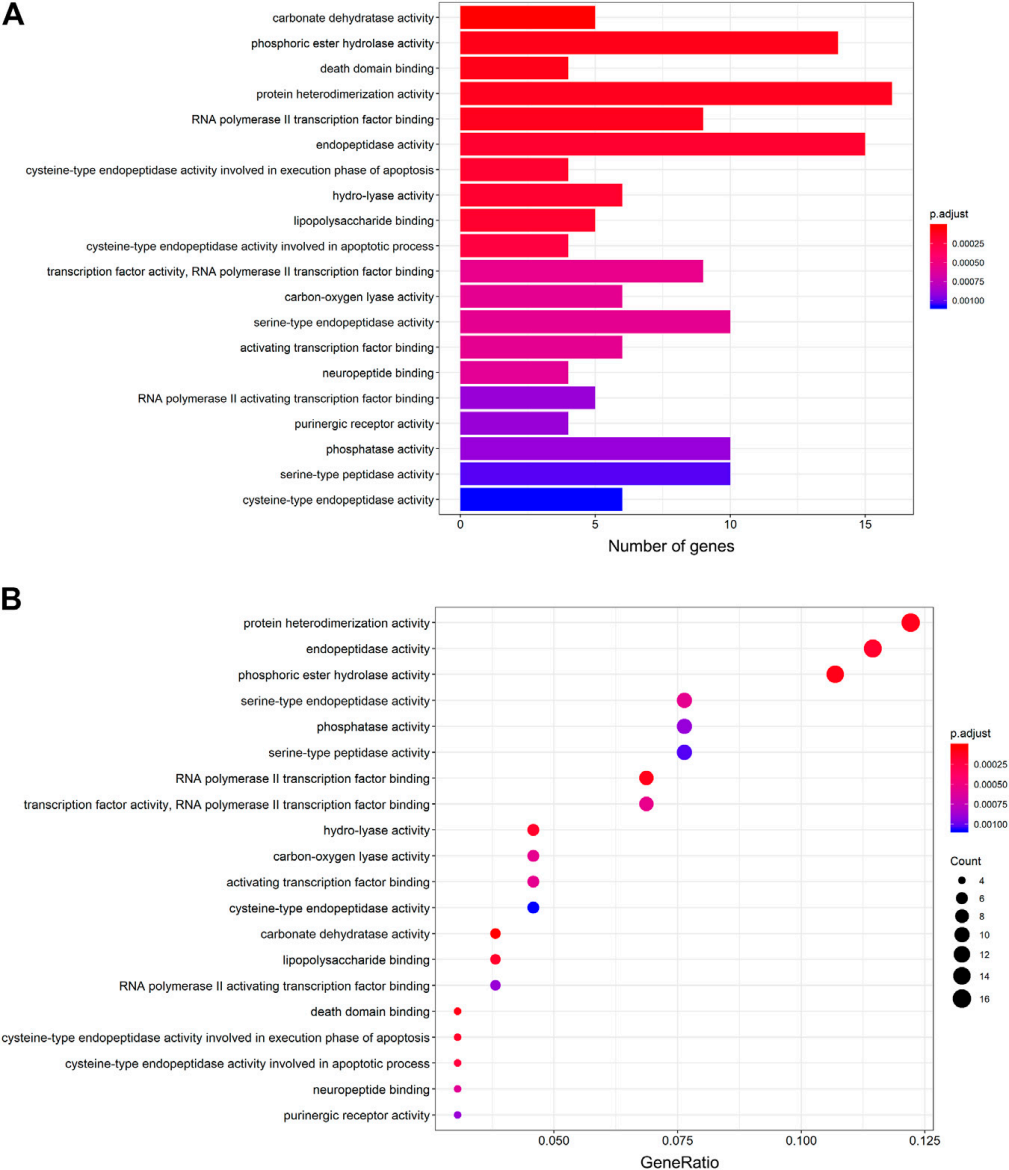
GO functional enrichment analysis of the active compounds of Cornus officinalis and Paeonia lactiflora. **(A)** Bar plot of top 20 enriched terms of GO functional analysis, ranked by enrichment score. The intensity of the colors represents the adjusted *p*-value. **(B)** Bubble plot displaying the top 20 enriched terms of GO functional analysis, ranked by gene count. The intensity of the colors represents the adjusted *p*-value and the bubble size corresponds to the number of genes.

**FIGURE 3:**
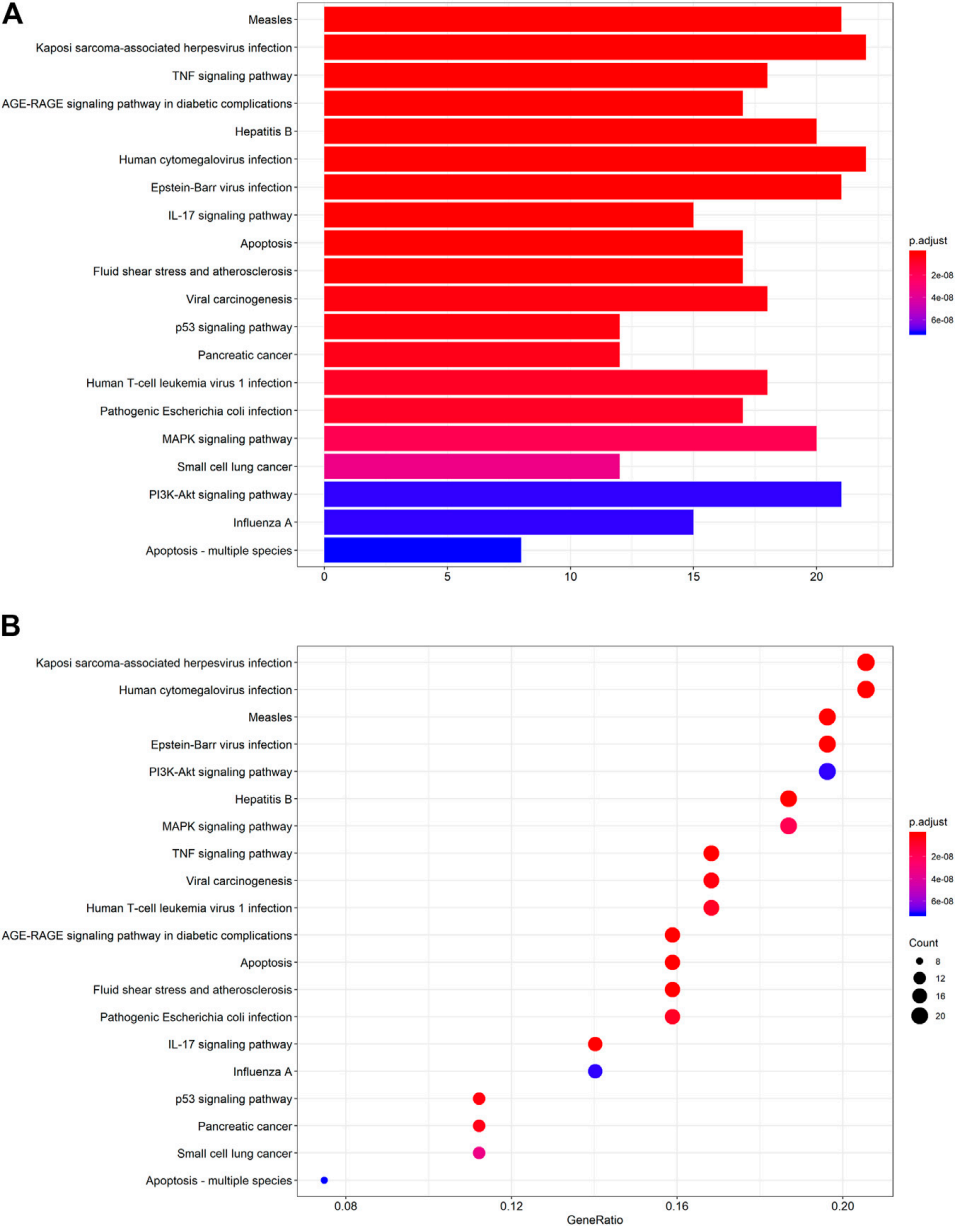
KEGG pathway enrichment analysis of the active compounds of Cornus officinalis and Paeonia lactiflora. **(A)** Bar plot of top 20 enriched pathways of KEGG analysis, ranked by enrichment score. The intensity of the colors represents the adjusted *p*-value. **(B)** Bubble plot displaying the top 20 enriched pathways of KEGG analysis, ranked by gene count. The intensity of the colors represents the adjusted *p*-value and the bubble size corresponds to the number of genes.

The protein targets of these compounds were predicted using three databases: Swiss, TCMSP, and PharmMapper (Figure 1B). In total, 132 unique target proteins were identified between the five compounds, with five common targets between Swiss and TCMSP. However, no target was identified in all three databases. The results of GO analysis revealed that the 132 target genes were significantly enriched in 93 GO terms, of which the top 20 terms based on adjusted *p*-value and gene count are shown in Figures 2A,B, respectively. The common terms found in the bar and bubble plots were identified and listed in order of gene count in Table 2. Among the eight GO terms, 52 unique target genes were identified, among which 12 (ATF2, CTSB, DDP4, FOS, IGHG1, JUN, KLK7, MMP1, MMP2, MMP3, MMP10, and PLAU) were found in three GO terms (the maximum). Similarly, the results of KEGG analysis revealed that the 132 target genes were significantly enriched in 118 signaling pathways. The top 20 KEGG terms based on adjusted *p*-value are shown in Figure 3A, and these terms were rearranged in order of gene count in Figure 3B. The top 10 terms in Figure 3B and their target genes are listed in Table 3. Among the ten KEGG terms, 46 unique target genes were identified, among which RELA and IKBKG were found in all ten KEGG terms. The next most enriched terms were TP53, which appeared nine times, and CASP3, IL6, and JUN, which each appeared eight times in the top ten KEGG terms.

**TABLE 2:** Target genes involved in top GO terms.

Id	Description	Target genes	Count
GO:0046982	Protein heterodimerization activity	ADORA2A, TYR, RELA, VEGFA, BCL2, BCL2L1, FOS, BAX, JUN, TP53, IKBKG, MCL1, ATF2, ADORA1, SMAD1, DRAP1	16
GO:0004175	Endopeptidase activity	F2, DPP4, PLAU, CTSB, CASP9, MMP2, CASP3, CASP8, MMP1, MMP3, MMP10, CASP1, KLK7, IGHG1, USP14	15
GO:0042578	Phosphoric ester hydrolase activity	EPHX2, PTPN6, PTPN1, INPPL1, ENPP7, PTPN2, PTPRF, ACP1, PDE4D, ADORA1, PPM1A, TAB1, PDE3B, DUSP6	14
GO:0004252	Serine-type endopeptidase activity	F2, DPP4, PLAU, CTSB, MMP2, MMP1, MMP3, MMP10, KLK7, IGHG1	10
GO:0016791	Phosphatase activity	EPHX2, PTPN6, PTPN1, INPPL1, PTPN2, PTPRF, ACP1, PPM1A, TAB1, DUSP6	10
GO:0008236	Serine-type peptidase activity	F2, DPP4, PLAU, CTSB, MMP2, MMP1, MMP3, MMP10, KLK7, IGHG1	10
GO:0001085	RNA polymerase II transcription factor binding	STAT3, FOS, JUN, TP53, CREB1, ATF2, RUVBL1, EXOSC9, SPEN	9
GO:0001076	Transcription factor activity, RNA polymerase II transcription factor binding	FOS, JUN, TP53, CREB1, ATF2, RORC, NR1D2, CITED2, SPEN	9

**TABLE 3:** Target genes involved in top KEGG terms.

Id	Description	Target genes	Count
hsa05167	Kaposi sarcoma-associated herpesvirus infection	RELA/STAT3/VEGFA/CCND1/FOS/BAX/CASP9/CDK4/JUN/IL6/CDK6/CASP3/TP53/MAPK8/PTGS2/IKBKG/CASP8/FGF2/ICAM1/CREB1/CSF2/EIF2AK2	22
hsa05163	Human cytomegalovirus infection	RELA/STAT3/VEGFA/CCND1/BAX/CASP9/CDK4/TNF/IL6/CDK6/CASP3/TP53/PTGS2/IKBKG/CASP8/IL1B/CREB1/PTGER3/PRKCG/ATF2/FASLG/MDM2	22
hsa05162	Measles	RELA/STAT3/CCND1/BCL2/BCL2L1/FOS/BAX/CASP9/CDK4/JUN/IL6/CDK6/CASP3/TP53/MAPK8/IKBKG/CASP8/IL1B/CCND2/FASLG/EIF2AK2	21
hsa05169	Epstein-Barr virus infection	RELA/STAT3/CCND1/BCL2/BAX/CASP9/CDK4/TNF/JUN/IL6/CDK6/CASP3/TP53/MAPK8/IKBKG/CASP8/ICAM1/CCND2/EIF2AK2/MDM2/TAB1	21
hsa04151	PI3K-Akt signaling pathway	RELA/VEGFA/CCND1/BCL2/BCL2L1/CASP9/CDK4/IL6/CDK6/TP53/IKBKG/FGF2/CREB1/MCL1/ATF2/NOS3/CCND2/FASLG/HSP90AA1/FGF1/MDM2	21
hsa05161	Hepatitis B	RELA/STAT3/BCL2/FOS/BAX/CASP9/TNF/JUN/IL6/CASP3/TP53/MAPK8/IKBKG/CASP8/CREB1/PRKCG/ATF2/BIRC5/FASLG/TAB1	20
hsa04010	MAPK signaling pathway	RELA/VEGFA/FOS/TNF/JUN/CASP3/TP53/MAPK8/IKBKG/FGF2/IL1B/PRKCG/ATF2/MAPK8IP2/FASLG/CD14/FGF1/PPM1A/TAB1/DUSP6	20
hsa04668	TNF signaling pathway	RELA/FOS/TNF/JUN/IL6/CASP3/MAPK8/PTGS2/IKBKG/CASP8/MMP3/ICAM1/IL1B/CREB1/SELE/ATF2/CSF2/TAB1	18
hsa05203	Viral carcinogenesis	RELA/STAT3/CCND1/BAX/CDK4/JUN/CDK6/CASP3/TP53/IKBKG/CASP8/CREB1/ATF2/CCND2/POLB/EIF2AK2/MDM2/GSN	18
hsa05166	Human T-cell leukemia virus 1 infection	RELA/CCND1/BCL2L1/FOS/BAX/CDK4/TNF/JUN/IL6/TP53/MAPK8/IKBKG/ICAM1/CREB1/ATF2/CSF2/CCND2/POLB	18

### Protein-Protein Interaction Network Analysis of Active Compounds

**FIGURE 4:**
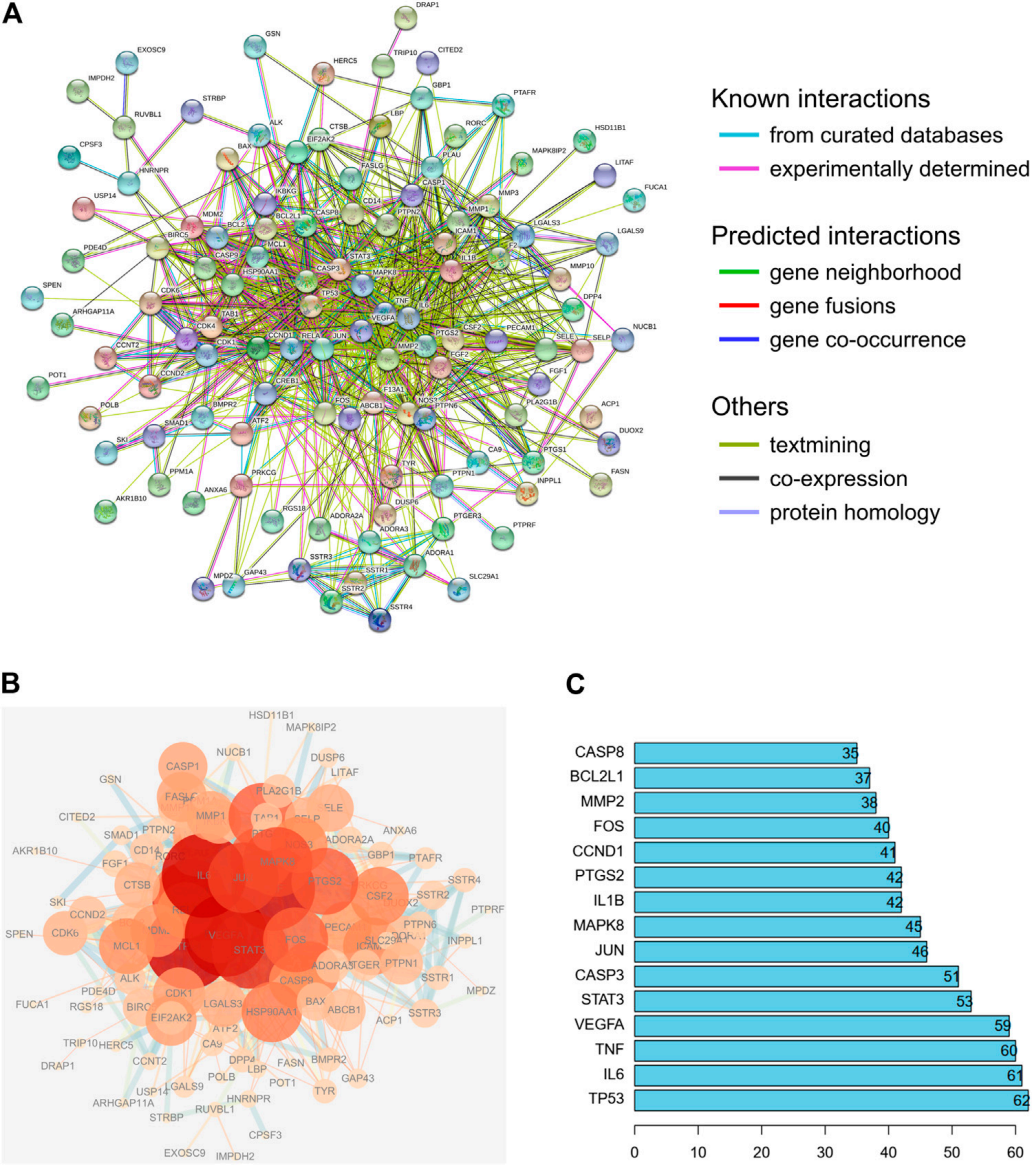
PPI network analysis of the identified targets of the active compounds of Cornus officinalis and Paeonia lactiflora. **(A)** The interactions among the target proteins of the selected compounds was mapped by constructing a PPI network to present the direct and indirect regulatory relationship between the targets. The nature of the interactions is differentiated using lines of different colors. **(B)** For visual analysis of the target proteins, a visual PPI network diagram was generated to evaluate the topology parameters of the network nodes. The size and darkness of the nodes represent the number of degrees of each node. The thickness and color of the edges represents the combination score, with yellow and blue indicating lower and higher combination scores, respectively. **(C)** Analysis of core genes, ranked by the number of times that each gene appears among relationship pairs within the network diagram.

To evaluate the relationship between the identified target proteins, a PPI network was constructed to present the direct and indirect regulatory interactions among the targets (Figure 4A). The nature of the interactions is differentiated using lines of different colors, as illustrated in Figure 4A. In addition, a visual PPI network diagram was generated to evaluate the topology parameters of the network nodes (Figure 4B). The size and darkness of the nodes represent the number of degrees of each node. The thickness and color of the edges represent the combination score, with yellow and blue indicating lower and higher combination scores, respectively. The core genes were further analyzed and ranked by the number of times that each gene appears among relationship pairs within the network diagram (Figure 4C). Among these interactions, there were 131 nodes and 913 edges in total, with an average node degree of 13.9. Nodes that are larger and darker in red are those with higher degrees of freedom (Figure 4B), suggesting that the represented genes are closely linked to other genes in the network and may be critically associated with RA pathogenesis. Among these, the top ten key genes in terms of degrees of freedom are TP53, IL6, TNF, VEGFA, STAT3, CASP3, JUN, MAPK8, IL1B, and PTGS2, with respective degrees of freedom of 62, 61, 60, 59, 53, 51, 46, 45, 42, and 42 (Figure 4C).

**FIGURE 5:**
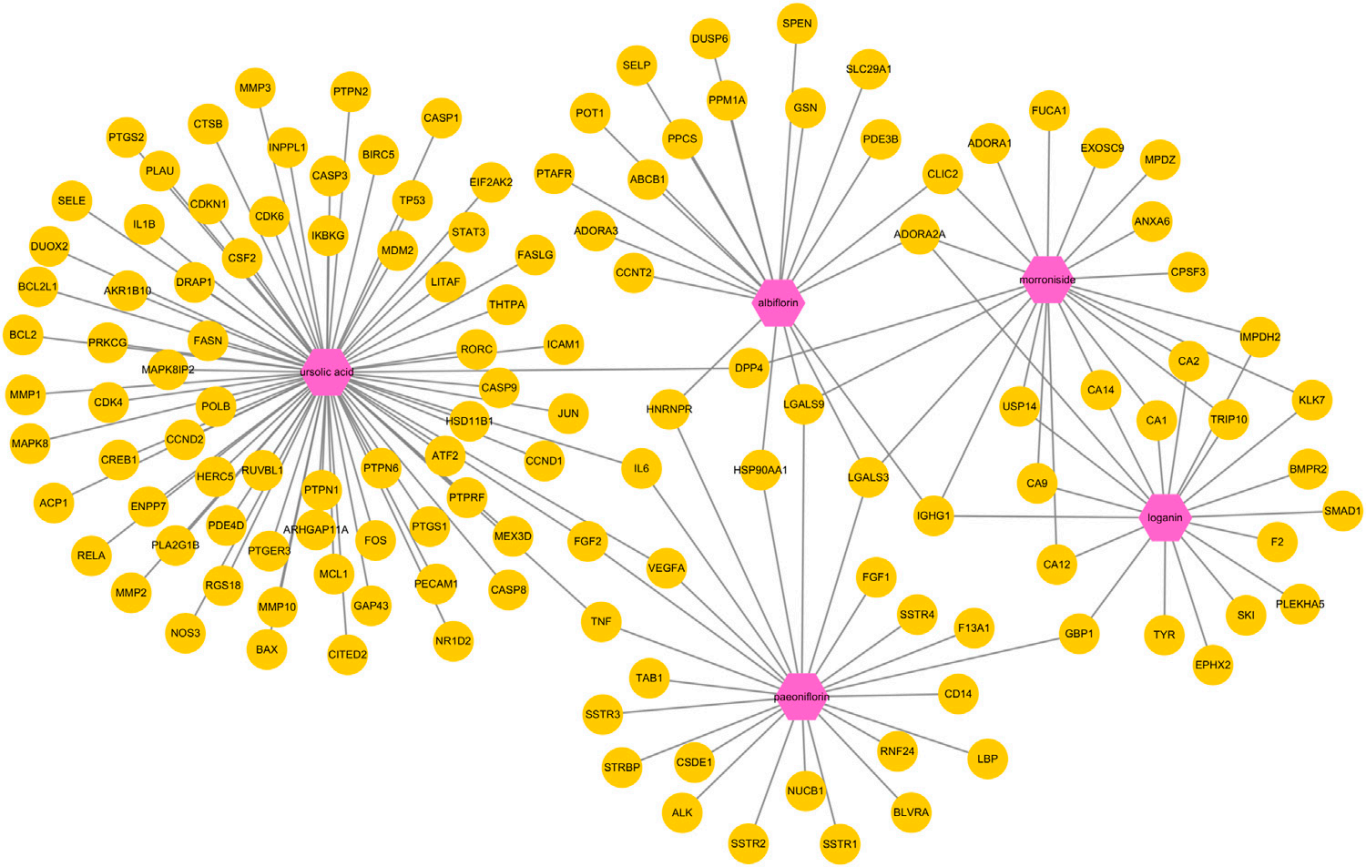
Pharmacological network map illustrating compound-targets relationships. The active compounds investigated in this study are displayed in pink hexagons and their targets are shown in yellow circles. Genes that are the target of two or more compounds are revealed by identifying the targets with multiple connections (degrees).

Next, a pharmacological network map was constructed to illustrate the interplay between compound-target relationships (Figure 5). The active compounds investigated in this study are displayed in pink hexagons and their targets are shown in yellow circles. Genes that are targeted by two or more compounds are revealed by identifying the nodes with multiple connections (Table 4). Among the identified targets, four (LGALS3, LGALS9, ADORA2A, and IGHG1) were the common targets of three compounds and 18 were the common targets of two compounds.

**TABLE 4:** Degrees of freedoms of genes targeted by multiple active compounds.

Compound/gene name	Degrees	Targeted by
Loganin	19	---
Ursolic acid	74	---
Paeoniflorin	24	---
Morroniside	21	---
Albiflorin	20	---
LGALS3	3	Morroniside, paeoniflorin, albiflorin
LGALS9	3	Morroniside, paeoniflorin, albiflorin
ADORA2A	3	Loganin, morroniside, albiflorin
IGHG1	3	Loganin, morroniside, albiflorin
HSP90AA1	2	Paeoniflorin, albiflorin
HNRNPR	2	Paeoniflorin, albiflorin
CLIC2	2	Morroniside, albiflorin
CA2	2	Loganin, morroniside
CA1	2	Loganin, morroniside
CA12	2	Loganin, morroniside
CA14	2	Loganin, morroniside
CA9	2	Loganin, morroniside
IMPDH2	2	Loganin, morroniside
KLK7	2	Loganin, morroniside
USP14	2	Loganin, morroniside
TRIP10	2	Loganin, morroniside
GBP1	2	Loganin, paeoniflorin
DPP4	2	Ursolic acid, morroniside
TNF	2	Ursolic acid, paeoniflorin
IL6	2	Ursolic acid, paeoniflorin
VEGFA	2	Ursolic acid, paeoniflorin
FGF2	2	Ursolic acid, paeoniflorin

### Common Target Genes of Rheumatoid Arthritis and Active Compounds: Prediction, Simulation, and Validation

**FIGURE 6:**
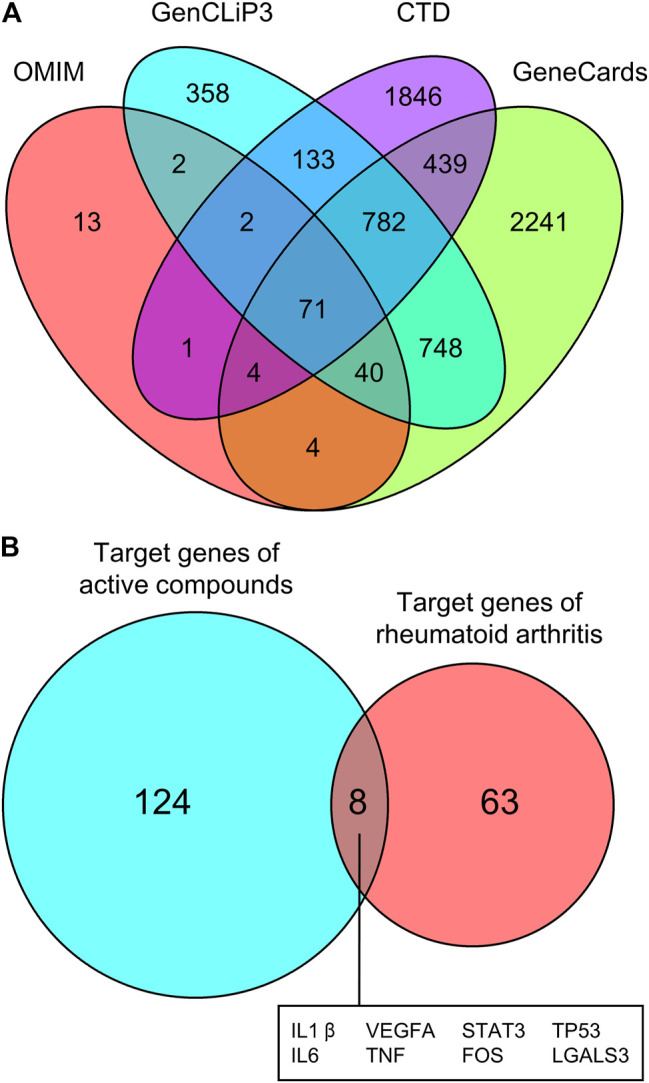
Common targets of RA and active compounds of Cornus officinalis and Paeonia lactiflora. **(A)** The gene targets of RA were screened using four databases (OMIM, GenCLip3, CTD, and GeneCards) and common targets between databases are illustrated using a Venn diagram. Among all identified targets, 71 were common between all four databases. **(B)** The 71 targets of RA were compared with the 132 previously identified targets of Cornus officinalis (loganin, morroniside, and ursolic acid) and Paeonia lactiflora (paeoniflorin and albiflorin) and a Venn diagram was generated to illustrate the number of common targets. Between RA and the active compounds, 8 common targets were identified.

We then screened for the target genes of RA in four databases: OMIM, GenCLiP3, CTD, and GeneCards. Among all of the identified targets, Venn diagram analysis revealed that there were 71 common targets of RA from these databases (Figure 6A). These 71 targets were compared with the 132 previously identified unique targets of the five active compounds (loganin, ursolic acid, morroniside, paeoniflorin, and albiflorin), revealing eight common targets (Figure 6B). These are namely IL1β, VEGFA, STAT3, TP53, IL6, TNF, FOS, and LGALS3.

**FIGURE 7:**
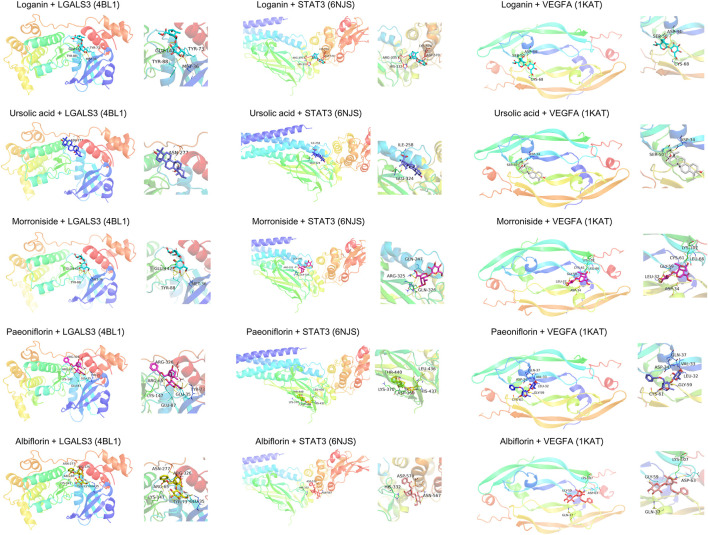
Molecular docking simulation of compound-target binding. Using AutoDock Vina, molecular docking analysis was performed between the five active compounds and three selected targets among the eight identified: LGALS3, STAT3, and VEGFA. The PDB IDs of the receptor proteins of LGALS3, STAT3, and VEGFA are 4BL1, 6NJS, and 1KAT, respectively. The three targets were able to spontaneously bind to each of the five active compounds via forces such as hydrogen bonds to form a stable conformation.

To elucidate the mechanism of compound-target binding, we evaluated the binding energy between the eight common targets and the five active compounds (Table 5). Lower binding energies correspond to stronger binding between compound and target (Aamir et al., 2018). Consistent with the compound-target relationships shown in Figure 5 and Table 4, the binding strengths between each identified target with their corresponding active compounds/compounds (bold values in grey cells in Table 5) are the highest among all active compounds. The exception was LGALS3, which was predicted to be targeted by morroniside, paeoniflorin, and albiflorin (Figure 5) but showed the greatest binding strength with ursolic acid, morroniside, and paeoniflorin. Using AutoDock Vina, we performed a molecular docking analysis between the five active compounds and three selected targets among the eight identified: LGALS3, STAT3, and VEGFA. The PDB IDs of the receptor proteins of LGALS3, STAT3, and VEGFA are 4BL1, 6NJS, and 1KAT, respectively. The results of molecular docking visualization (Figure 7) showed that the three targets were able to spontaneously bind to each of the five active compounds, via forces such as hydrogen bonds to form a stable conformation.

**TABLE 5:** Binding energy between selected targets and active compounds investigated in this study.

		Binding energy (kcal/mol)				
Target	Structure	Loganin	Ursolic acid	Morroniside	Paeoniflorin	Albiflorin
**IL1B**	1T4Q	−6.6	−**7.7**	−6.9	−6.7	−6.8
**IL6**	IIL6	−6.6	−**7.8**	−6.6	−**7.2**	−7
**VEGFA**	1KAT	−6.8	−**7.7**	−6.3	−**7.6**	−7.3
**TNF**	1A8M	−6.5	−**7.8**	−6.7	−**7.2**	−7
**STAT3**	6NJS	−7.4	−**8.2**	−7.2	−7.2	−7.4
**FOS**	1G2E	−7	−**7.7**	−7.1	−7.4	−7.4
**TP53**	6MXZ	−6.8	−**7.5**	−6.3	−7.4	−6.5
**LGALS3**	4BL1	−8.2	−8.6	−**8.8**	−**8.4**	−**8.3**

Note: Grey cells with values in bold correspond to the compound-target relationship predicted in Figure 5.

### Experimental Validation of Target Protein Expression in Collagen-Induced Arthritis Rat Model

**FIGURE 8:**
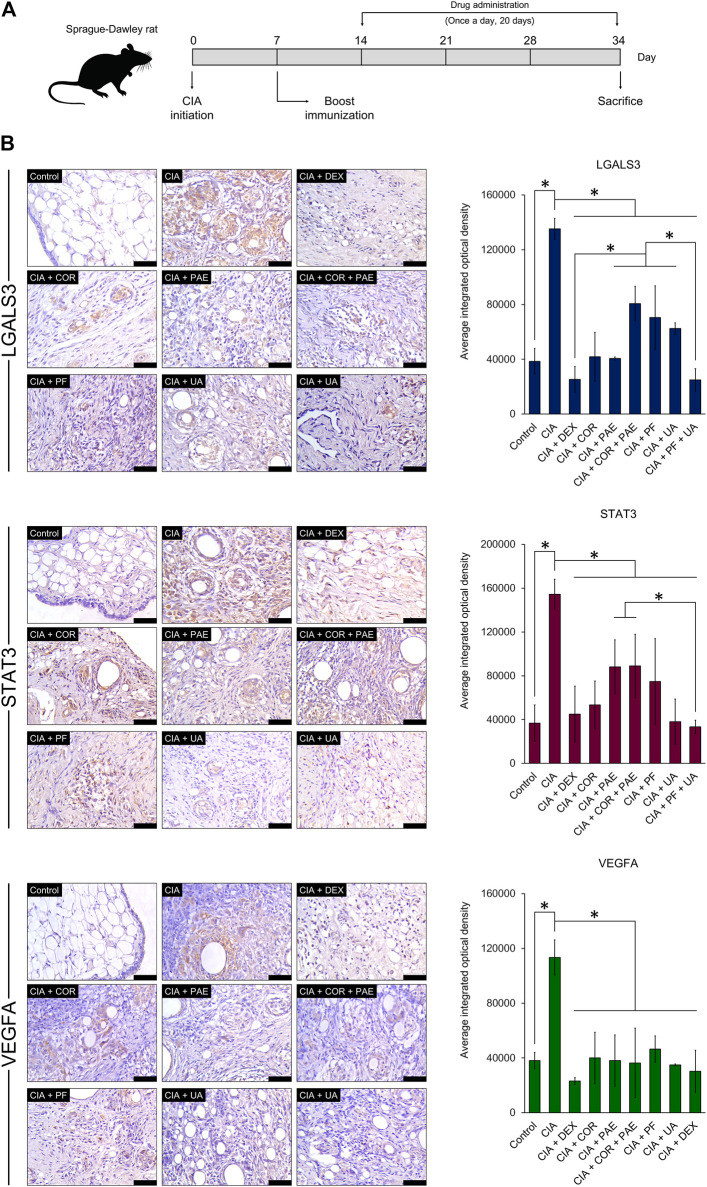
Experimental validation of target protein expression in CIA rat model. **(A)** Sprague-Dawley rats were induced by CIA and treated with Cornus officinalis (COR, 3.36 g/kg/d) or/and Paeonia lactiflora (PAE, 6.27 g/kg/d), paeoniflorin (PF, 7.5 mg/kg/d) or/and ursolic acid (UA, 25 mg/kg/d), or dexamethasone (DEX, 0.5 mg/kg/d) daily for 20 days. **(B)** After sacrifice, synovial tissues were isolated from the rats and immunohistochemical staining was performed to assess the positive expression (brown areas in images) of LGALS3, STAT3, and VEGFA. Scale bar = 50 μm. Areas of positive staining were quantified using ImagePro Plus. The data are expressed as the average integrated optical density ± standard deviation (*n* = 3). * indicates *p* < 0.05.

For a preliminary *in vivo* experimental validation of the predicted compound-target relationship, we evaluated the expression of LGALS3, STAT3, and VEGFA in a rat model of CIA (Figure 8A). Successful establishment of the CIA model was confirmed by assessing the arthritic score and swelling degree in the rats throughout the experimental period, as well as histological verification (data not shown). After the induction of CIA, Sprague-Dawley rats were subjected to daily treatment of Cornus officinalis (COR) or/and Paeonia lactiflora (PAE), their respective active compounds ursolic acid (UA) or/and paeoniflorin (PF), or dexamethasone (DEX) as a positive control (Cohen et al., 1960). UA and PF were selected for these experiments because they had previously been identified as the compounds with the most drug-like pharmacological properties. After 20 days of treatment, synovial tissues were isolated from the experimental rats and the synovial expression of LGALS3, STAT3, and VEGFA was assessed by immunohistochemistry (Figure 8B). Upon CIA induction, we noticed a significant increase in the positive synovial expression of LGALS3, STAT3, and VEGFA compared to that in the Control group (*p* < 0.05). The administration of COR or/and PAE, UA or/and PF, or DEX significantly reduced the expression of the three proteins in the synovial tissues of CIA-induced rats. In terms of VEGFA expression, the difference between the treatments was not significant. However, the effect of PF + UA and DEX was significantly better than that of all other treatments (except COR alone) in downregulating LGALS3 expression. Furthermore, PF + UA exerted a stronger effect in downregulating STAT3 expression than PAE and COR + PAE. Collectively, these results signify that the expression of LGALS, STAT3, and VEGFA, which can be considered as tissue biomarkers of RA, act as indicators of the effectiveness of treatment using various compounds.

## Discussion

Medicinal ingredients in TCM are composed of hundreds and even thousands of active chemical compounds. Each compound interacts with a variety of genes, proteins, and molecular pathways within a biological system, giving rise to the multi-target characteristic of TCM (Li et al., 2012). Consequently, the same type of TCM may exert diverse therapeutic effects in different diseases and models. For example, Cornus officinalis has been shown to promote caspase-mediated apoptosis of triple-negative breast cancer cells (Telang et al., 2019), while also exerting therapeutic effects against diabetic nephropathy via one of its active compounds, loganin (Ma et al., 2014). Similarly, the anti-inflammatory and immunomodulatory properties of Paeonia lactiflora have been widely recognized (He and Dai, 2011), but it also reportedly exhibited anti-oxidative and neuroprotective activity (Lee et al., 2008). The complexity of the interaction between active compounds and their targets has thus prompted the emergence of specific methods of accurately and rapidly screening and identifying relevant players in disease progression (Wang et al., 2012).

To this end, systems and network pharmacology offers a simple and efficient solution to the analysis of active compounds and targeting mechanisms using large-scale databases and screening tools. The main advantage and purpose of using network pharmacology is that it provides a systematic and objective analysis of the molecular roles of our selected compounds. This is done through a variety of databases and bioinformatic resources, giving us a direction towards future research by identifying specific targets of interest that can be further explored experimentally. The underlying mechanism of Cornus officinalis and Paeonia lactiflora has been predicted using network pharmacology in association with diseases such as ulcerative colitis (Zhang et al., 2019), Alzheimer’s disease (Zeng et al., 2019), Parkinson’s disease (Du et al., 2020), and depression (Liu et al., 2020). Using a variety of network pharmacology resources, we analyzed the potential molecular functions of Cornus officinalis and Paeonia lactiflora pertaining to RA by breaking down the properties of their individual active compounds. We found that the active compounds of Cornus officinalis (loganin, ursolic acid, and morroniside) and Paeonia lactiflora (paeoniflorin and albiflorin) included in this study share eight common target genes with RA, namely IL1β, VEGFA, STAT3, TP53, IL6, TNF, FOS, and LGALS3. Using molecular docking simulation, we showed the binding of each active compound with three chosen targets (LGALS3, STAT3, and VEGFA). We finally verified the targeting effect of ursolic acid and paeoniflorin, the most “drug-like” active compounds of Cornus officinalis and Paeonia lactiflora, respectively, in a rat model of CIA. The results of immunohistochemical staining revealed that CIA induced a significant increase in the synovial expression of LGALS3, STAT3, and VEGFA. The protein expression of these targets was subsequently attenuated by Cornus officinalis or/and Paeonia lactiflora, as well as ursolic acid or/and paeoniflorin, confirming the proposed compound-target relationships.

GO and KEGG analysis enable the identification of key pathways and genes involved in disease development and progression. Herein, we analyzed the top eight GO terms and top ten KEGG terms pertaining to the five active compounds selected for our study. In the GO analysis (Table 2), three common targets between active compounds and RA appeared in two terms: “protein heterodimerization activity” (VEGFA, FOS, and TP53) and “RNA polymerase II transcription factor binding” (STAT3, FOS, and TP53), both belonging to the “molecular function” category. KEGG analysis revealed three specific molecular signaling pathways among the ten most enriched terms, namely PI3K-Akt, MAPK, and TNF signaling pathway (Table 3). Respectively, these pathways contain three (VEGFA, IL6, and TP53), five (VEGFA, FOS, TNF, TP53, and IL1B), and four (FOS, TNF, IL6, and IL1B) target genes that are found in our eight key common targets. We note that each target appears twice within the pathways, but among them, only three (TNF, IL6, and VEGFA) are targeted by more than one active compound. Interestingly, all three are targets of both ursolic acid and paeoniflorin (Table 4), which have been identified as the most “drug-like” substances in this study. Collectively summarizing these evidences, we suggest that Cornus officinalis and Paeonia lactiflora mainly exert their therapeutic effects against RA by targeting TNF, IL6, and VEGFA via modulation of PI3K-Akt, MAPK, and TNF signaling.

Among the eight common targets between active compounds and RA, LGALS3, STAT3, and VEGFA were selected for further validation based on a comprehensive evaluation of their degrees of freedom and binding energies. These three targets have important implications in association with RA. LGALS3 reportedly contributed to RA development by promoting inflammation (Mendez-Huergo et al., 2018) and activating synovial fibroblasts (Ohshima et al., 2003). The specific effects of ursolic acid and paeoniflorin on the expression of LGALS3 has not been investigated in the literature. Nevertheless, a targeting relationship between ursolic acid and the tyrosine-protein kinase MER, of which LGALS3 is a ligand, has been suggested (He et al., 2015), and a binding relationship between paeoniflorin and LGALS3 has been predicted by systems pharmacology (Chen et al., 2020). STAT3 has been shown to be required for synoviocyte survival in RA (Krause et al., 2002), and STAT3 inhibitors have been proposed as therapeutic candidates for RA treatment (Oike et al., 2017). Ursolic acid has been shown to suppress colon cancer (Wang et al., 2013) and hepatocellular carcinoma (Liu et al., 2017) by inhibiting STAT3 signaling, whereas paeoniflorin exerted anti-diabetic effects (Li et al., 2018) and suppressed glioma cell growth (Nie et al., 2015) by targeting STAT3. The angiogenic factor VEGFA is produced by active synovial fibroblasts and has been implicated in the pathogenesis of RA, being one of its most important biomarkers (Taylor, 2002). Studies have revealed the chemosensitizing effects of ursolic acid in colon cancer (Shan et al., 2016) and the protective effects of paeoniflorin against oxidative injury (Song et al., 2017), both via suppression of VEGF signaling. Taken together, our results are in agreement with previous research showing the compound-target relationship between ursolic acid/paeoniflorin and each of LGALS3, STAT3, and VEGFA. Our immunohistochemical studies in a rat CIA model preliminarily validated these compound-target relationships. However, the significance of these relationships in RA have not been elucidated and require further exploration.

## Conclusion

Our study presents a comprehensive analysis of the active compounds of Cornus officinalis and Paeonia lactiflora based on network pharmacology and molecular docking. This was complemented by a preliminary immunohistochemical verification of the identified compound-target relationships in a rat model of CIA. We systematically revealed the pharmacological and molecular roles of Cornus officinalis and Paeonia lactiflora, further establishing them as important candidate drugs in the treatment and management of RA. One limitation of this study was that the analysis prompted us to focus on ursolic acid and paeoniflorin as the main active compounds of Cornus officinalis and Paeonia lactiflora, respectively, in RA treatment. This was solely based on evaluation of “drug-likeness”, which is one of the criteria of assessing pharmacological activity but certainly not the only one. The roles of the other active compounds (loganin, morroniside, and albiflorin) did not receive much attention in this study, but will form the basis of follow-up investigation on the role of Cornus officinalis and Paeonia lactiflora in RA therapy. In addition, the role of PI3K-Akt, MAPK, and TNF signaling in the treatment of RA by Cornus officinalis and Paeonia lactiflora was not verified experimentally in this study and remains to be explored in follow-up research.

## Data Availability

The raw data supporting the conclusions of this article will be made available by the authors, without undue reservation.
