# Supplementary material for: Evaluating the Therapeutic Mechanisms of Selected Active Compounds in Cornus Officinalis and Paeonia Lactiflora in Rheumatoid Arthritis via Network Pharmacology Analysis
Source: Front Pharmacol. 2021 Apr 21;12:648037. doi: 10.3389/fphar.2021.648037 (PMC8097135; doi:10.3389/fphar.2021.648037)
Supplement: Supplementary file 1 [file table1.docx]

**Supplementary Materials**

**Evaluating the therapeutic mechanisms of selected active compounds in Cornus officinalis and Paeonia lactiflora in rheumatoid arthritis via network pharmacology analysis**

**Qinglin Li^1,2^, Shaoqi Hu^1^, Lichuang Huang^1^, Jida Zhang^3,*^, Gang Cao^1,*^**

^1^School of Pharmacy, Zhejiang Chinese Medical University, Hangzhou, China

^2^The Cancer Hospital of the University of Chinese Academy of Sciences (Zhejiang Cancer Hospital), Institute of Basic Medicine and Cancer, Chinese Academy of Sciences, Hangzhou, China

^3^Institute of Basic Research in Clinical Medicine, College of Basic Medical Science, Zhejiang Chinese Medical University, Hangzhou, China

**^*^Correspondence**

Gang Cao
cgang1126@163.com

Jida Zhang
zhjd82@tom.com

**Table S1. Common gene targets of rheumatoid arthritis between OMIM, GenCLiP3, CTD, and GeneCards database.**

| IL1B |
| --- |
| IFNG |
| IL6 |
| IL10 |
| VEGFA |
| STAT1 |
| STAT4 |
| IL18 |
| TNFAIP3 |
| IL6ST |
| HLA-DRB1 |
| NCF1 |
| MIF |
| TRAF1 |
| SLC11A1 |
| IRF5 |
| SLC22A4 |
| IL2RB |
| CTLA4 |
| PTPN22 |
| NFKBIL1 |
| PADI4 |
| IL12A |
| CD244 |
| TNF |
| IL1RN |
| STAT3 |
| IL17A |
| FOS |
| TNFRSF1A |
| CD44 |
| PLA2G4A |
| CDKN1A |
| TP53 |
| S100A9 |
| SPP1 |
| IL3 |
| STAT6 |
| LGALS3 |
| CXCL12 |
| PTGES |
| HLA-C |
| TBX21 |
| SAA1 |
| SOCS3 |
| HLA-G |
| BMP2 |
| NOD2 |
| MAPKAPK2 |
| PSMB8 |
| NLRP3 |
| CCR2 |
| GUSB |
| PLA2G2A |
| DKK1 |
| CCR5 |
| PDGFB |
| SERPINH1 |
| MMP14 |
| IL2RG |
| FSTL1 |
| IL33 |
| FLT1 |
| MAP3K14 |
| ADAM17 |
| IL1RL1 |
| C5 |
| TNFRSF9 |
| NR4A2 |
| TNFRSF1B |
| TRPC1 |
